# Use of Multispectral Microscopy in the Prediction of Coated Halftone Reflectance

**DOI:** 10.3390/jimaging8090243

**Published:** 2022-09-08

**Authors:** Fanny Dailliez, Mathieu Hébert, Lionel Chagas, Thierry Fournel, Anne Blayo

**Affiliations:** 1 Univ Grenoble Alpes, CNRS, Grenoble INP (Institute of Engineering Université Grenoble Alpes), LGP2, F-38000 Grenoble, France; 2 Univ Lyon, UJM-Saint-Etienne, CNRS, Institut d’Optique Graduate School, Laboratoire Hubert Curien UMR 5516, F-42023 Saint-Etienne, France

**Keywords:** halftone colors, optical models, color image, hyperspectral imaging, spectral reflectance, coating, microscopy

## Abstract

When a print is coated with a transparent layer, such as a lamination film or a varnish layer, its color can be modified compared to the uncoated version due to multiple reflections between the layer-air interface and the inked substrate. These interreflections involve a multiple-convolution process between the halftone pattern and a ring-shaped luminous halo. They are described by an optical model which we have developed. The challenge at stake is to observe the impact of the coated layer on the print spectral reflectances and see if it can be predicted. The approach is based on pictures of the print captured with a multispectral microscope that are processed through the optical model to predict the spectral pictures of the coated print. The pictures averaged on the spatial dimension led to spectral reflectances which can be compared with macroscale measurements performed with a spectrophotometer. Comparison between macroscale measurements and microscale measurements with a multispectral microscope being delicate, specific care has been taken to calibrate the instruments. This method resulted in fairly conclusive predictions, both at the macroscale with the spectral reflectances, and at the microscale with an accurate prediction of the blurring effect induced by the multi-convolutive optical process. The tests carried out showed that the optical and visual effect of a coating layer on single-ink or multi-ink halftones with various patterns can be predicted with a satisfactory accuracy. Hence, by measuring the spatio-spectral reflectance of the uncoated print and predicting the spatio-spectral reflectance of the coating print, we can predict the color changes due to the coating itself. The model could be included in color management workflows for printing applications including a finishing coating.

## 1. Introduction

**Figure 1:**
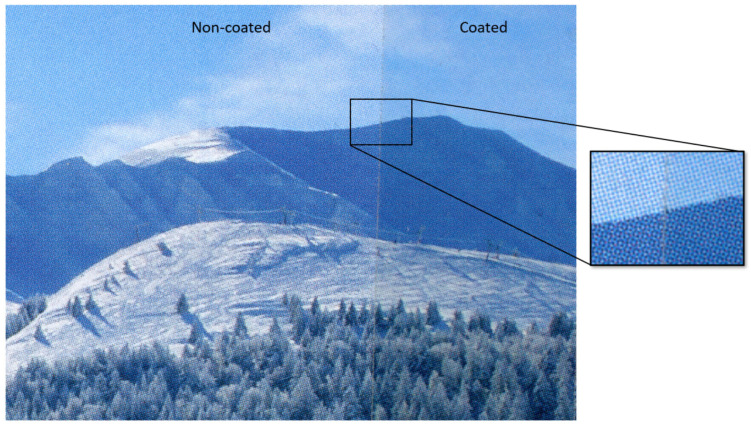
Scanned picture from a printed magazine [22], the right part was coated with a tape layer.

At a reading distance, the color of a print may look uniform, but its color is actually generated by microscopic dots of primary color inks [1]. The eye resolution does not allow to perceive these dots, but they are clearly visible when observed with a microscope, as can be seen in [2]. Colors reproduced by doted areas, called *halftone colors*, can then be uniformly colored at the macroscale and non-homogeneously colored at the microscale. When a non-homogenously colored substrate is coated with a smooth transparent layer, inter-reflections occur inside the transparent layer which can modify the macroscopic color of the print [3,4,5,6,7,8]. These inter-reflections are halo-shaped, a phenomenon thoroughly explained in previous publications [9,10,11,12,13,14], which is caused by the angle dependency of the Fresnel reflectance at the coating-air interface. This phenomenon is referred to as “halation” in the literature specific to photography at the turn of the 20th century, even though the term covers different types of optical phenomena that can lead to a blurring effect in imaging. In a coated print, this halo effect enables light to transit from one small colored area to another one, which increases the probability for light to meet colored areas and be absorbed by them. It tends to darken the macroscopic reflectance of halftone prints coated with a smooth transparent layer, which can have a strong impact on color management in the printing domain. This impact is illustrated Figure 1, where a printed halftone picture is overlayed on the right side with a clear adhesive film. This results in a darkening of the colors in the coated area due to the halo effect, a color change that we would like to predict in order to ease, in the future, color management systems for printing when a coating is used. 

The aim of this paper is to show that we can predict the spectral reflectance (and thereby the color) of coated microscopic halftones from the spectral reflectance of the non-coated halftones and from the thickness of the coating layer. The study was already conducted on macroscale halftones composed of lines, predictions and experiments showed a good agreement [14]. The main challenge of the current study is to deal with microscopic 2D halftones. Indeed, at this scale, the halftone reflectances are no longer separated in inked and non-inked areas but, on the contrary, reflectances vary continuously from one colored area to another one due to an optical phenomenon called *optical dot gain* [15,16,17,18,19]. This phenomenon is caused by light propagation inside the substrate and results in a blur of the edges of the inked dots and a modification of the spatial reflectances of the print. Optical models like Clapper-Yule or Yule-Nielsen models enable to predict the macroscopic color of halftone prints with optical dot gain. However, they are based on macroscale reflectance measurements and do not describe the spatial reflectance of halftones [20,21]. The halo effect has a spatial impact on the halftone reflectances which cannot be taken into account through macroscale measurements. A specific apparatus was then designed to measure the microscopic spectral and spatial reflectances of the halftones. It is presented in Section 2 along with the optical model adapted to microscale halftones. An experiment was conducted to verify the model and evaluate the impact of the coating layer. The results are displayed in a Section 3.

## 2. Materials and Methods

**Figure 2:**
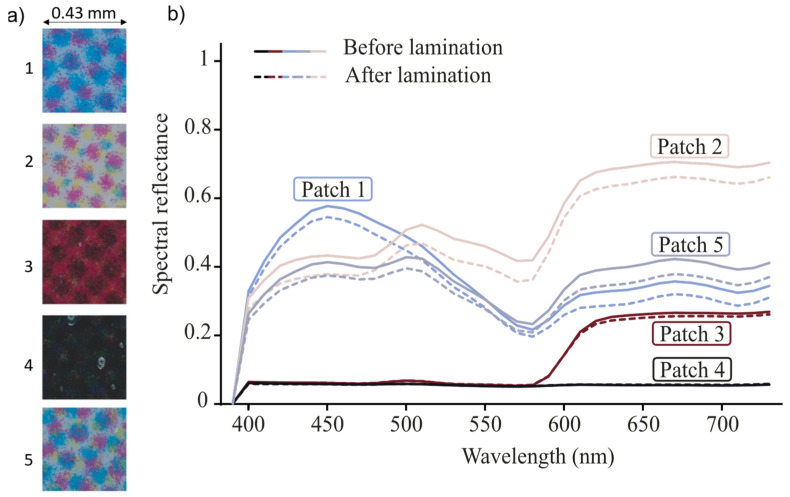
Example of halftones and impact of a coating layer on their reflectance. (**a**) Images of coated halftone patches with a microscope. (**b**) Spectrophotometric measurements of their reflectance before and after coating with a lamination film.

The darkening phenomenon due to the halo effect is visible in Figure 2 where the spectral reflectances of patches of a halftone print were measured before and after coating. The spectral reflectance measurements were made with the spectrophotometer CM-2600d from Konica Minolta, 8 mm aperture, with a di:8° geometry and including a UV filter to prevent the fluorescence of optical brighteners contained into the support. The coating was a 25 µm thick bright lamination film coated with the laminator DRY 350 W from RBS. In this picture, reflectances decrease when the halftones are overlaid with a transparent layer. Only the reflectance of patch 4 is not impacted by the coating layer as the print is almost uniformly colored at the microscopic scale in this area. 

To evaluate this darkening, a microscopic reflectance measurement system was developed, presented below. 

### 2.1. Multispectral-Imaging Microscope

**Figure 3:**
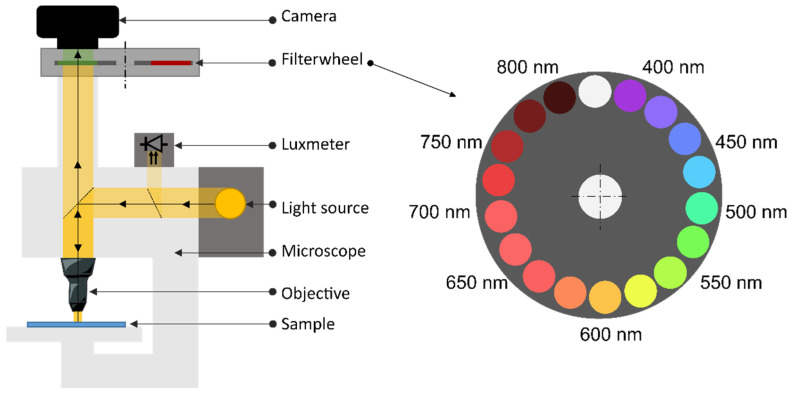
Scheme of the setup. (Imaging optics inside the microscope are not displayed).

In order to characterize the spectral reflectances of halftone prints at the microscopic scale, a custom multispectral imaging microscope was developed. The hardware, described in [23], is composed of a microscope, colored optical filters mounted on a wheel, and a commercial digital camera. The camera captures an RGB image through each optical filter of a wheel. The filters are of small wavelength bandwidth, which allows to reconstruct the multispectral reflectance of the sample for various wavelengths. The scheme of the setup is displayed in Figure 3.

The setup is composed of a microscope Zeiss Axio Imager M1 m mounted with the objective EC Epiplan Neofluar 5×/0.13 HD DIC. The light source is a Zeiss HAL 100 tungsten-halogen lamp set at 3200 K; it was turned on at least 20 minutes before the experiments to reach the steady state. Its emission spectrum is stronger for large wavelengths than for small wavelengths. To remove the specular reflections on the sample, a polarizer and an analyzer are used in a crossed configuration inside the microscope. A luxmeter, Yocto-Light-V3 LIGHTMK3 from Yoctopuce, is inserted across the path of the incident light beam in order to calibrate the irradiance of the sample for each image capture. The photodiode, manufactured by Rhoms (reference BH1751FVI), can measure in the range of 0 to 100,000 lux with a resolution of 0.25 lux. The camera is a commercial reflex camera, Canon 1200D with a RGGB CMOS sensor. The ISO was set to 800. The sensor, coded in 14 bits, has a resolution of 3516 × 5344 pixels. The camera is remotely monitored by a computer through a Python program. The filterwheel is composed of 17 filters (Hard Coated OD 4.0 25 nm Bandpass Filters from Edmund Optics) and driven by a motor. The camera captures at least one picture per filter with an adapted exposure time. The central wavelengths of the filters are in the range [400; 800 nm] with a 25 nm step. The filters have a 25 nm bandwidth. In practice, 13 filters are used in the range [400; 700 nm] corresponding to the spectral bandwidth of the light source.

To retrieve the spatial and spectral reflectances of a sample from the pixel values obtained through the camera, the apparatus is calibrated as follows. One pixel value is related to reflectance through [24,25,26,27]:

(1) $$V=Te\int_{\lambda }E\left(\lambda \right)F\left(\lambda \right)B\left(\lambda \right)s\left(\lambda \right)R\left(\lambda \right)d\lambda +V_{k}$$

where *V* is the pixel value associated with one optical filter, *T* is the exposure time (it has been verified that *V* is linear with *T*), *e* is proportional to the irradiance on the sample, measured with the photodiode, *E* is the spectral power distribution of the light source, *F* is the spectral transmittance of the filter, *B* is the spectral transmittance of the bayer filter, *s* is the spectral response of the sensor, *R* is the reflectance of the sample associated with the considered pixel, *λ* is the wavelength, and *V_k_* is a constant corresponding to the pixel value in the dark. *T* is monitored by the computer and can be retrieved in the metadata of the pictures.

*E*, *F*, *B*, and *s* are intrinsic to the device and complex to measure as they can depend on the spatial position of the pixel. If *R* is constant within the spectral bandwidth of the filter, Equation (1) becomes:

(2) $$V=R\left(\lambda^{\prime }\right)TeQ+V_{k}$$

where

(3) $$Q=\int_{\lambda }E\left(\lambda \right)F\left(\lambda \right)B\left(\lambda \right)s\left(\lambda \right)d\lambda$$

and *R(λ′)* is the reflectance of the sample at the central wavelength of the filter, *λ′*. To calibrate the system, *V_k_* and *Q* need to be determined.

During the calibration process, the value of each pixel of the camera in the dark, *V_k_*, was determined once forever by capturing pictures in the dark for each exposure times. The results, for each exposure time, were averaged over 9 measurements and were used afterward to calibrate all measurements. The average pixel value in the dark was 2047 ± 11.

The measurement of *Q* was made for each series of measurement through the capture of an opal glass spectralon (Commission of the European communities, community bureau of reference BCR, reference material number 406, individual identification number 0004) [28]. *Q* was derived, for each filter:

$$Q=\frac{V-V_{k}}{R_{c}\left(\lambda^{\prime }\right)Te}$$

where *R_c_* is the spectral reflectance of the opal glass spectralon at the central wavelength of the considered filter. This reflectance was determined through a measurement with a spectrophotometer, CM-2600d from Konica Minolta, with a di:8° geometry, including a UV cutoff filter, and an aperture of 8 mm diameter. It was measured 10 times and showed negligible uncertainties. For one filter, the reflectance at the central wavelength, *R_c_*, was the average reflectance over the 25 nm bandpass of the filter. The specular component of this spectrophotometric measurement (which is not included in the microscopic measurement) was removed by subtracting 0.04 to *R_c_*. The entire process was repeated four times and the final *Q* value is the average of the four computed *Q* values.

At this point, the apparatus is calibrated. The reflectance of any sample can then be determined from its pixel value *V*:

(4) $$R\left(\lambda^{\prime }\right)=\frac{V-V_{k}}{TeQ}$$

As the response of the microscope was low for certain pixels of the bayer matrix according to the studied wavelength (for instance red bayer filter coupled with a blue optical filter), one pixel of the Bayer matrix was selected for each considered filter of the filterwheel, dividing the resolution of the pictures by 2 in each dimension. Moreover, the non-imaging part of the sensor was cropped. In the end, the reflectance pictures were 1728 × 2592 pixels, with an imaging resolution of 1.386 µm per pixel. Sensor saturation was prevented thanks to high dynamic range measurements (HDR) where images were captured twice with different exposure times. The accepted pixel value range, once *V_k_* removed, was [100; 12,000], out of a range of approximately [0; 14,000]. Pixels still out of this range after the HDR recombination were discarded from the calculations, as well as all reflectance above 1. In practice, in all calculations, they were considered as Not a Number (NaN), except when displayed in pictures where they were set equal to 1 to outline them, and in convolution operations where they were set equal to the average spectral and spatial reflectance of the sample. During experiments, NaNs were largely avoided and showed negligible impacts on the results.

**Figure 4:**
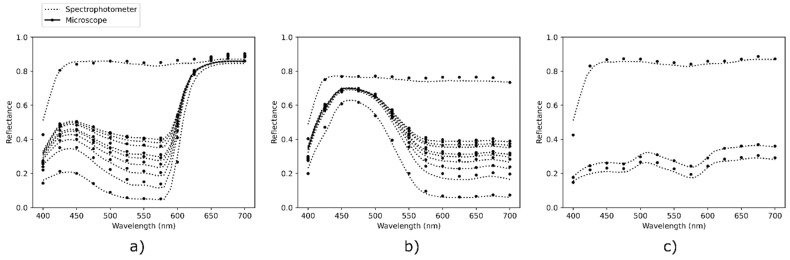
Average reflectance of the multispectral microscope images, compared with macroscale reflectance measurement with a spectrophotometer. (**a**) Magenta line halftones, (**b**) cyan line halftones, (**c**) multicolored dot halftones.

Using the spectrophotometer in the calibration process enables to compare the microscopic measurements from the custom apparatus (of resolution 1.386 µm per pixel) with the macroscopic ones from the spectrophotometer (measuring on an area of 8 mm diameter). Various halftone colors were designed and printed according to the protocol detailed in the Section 2.3, and their reflectances were captured with the two systems. The average spectra of the images were calculated and compared with the spectrophotometric measurements, displayed in Figure 4. A contribution of 0.04 was added to all reflectances which were measured with the microscope to account for the specular reflection included in the spectrophotometric measurement [29]. The results showed a good agreement between the two measuring methods. The color difference between spectrophotometric and microscopic measurement was calculated with the $\Delta E_{00}^{*}$ determined with the 2° observer, illuminant D65, and a perfect reflector as white reference. It is in average equal to $\Delta E_{00}^{*}=1.00\pm 0.47$ unit, which shows the uncommonly good agreement between the spectrophotometric and the microscopic measurements, in particular as they have different measurement geometries. Detailed performances are displayed in Table in Section 3. 

Small fluctuations in the reflectance measurements with the microscope were observed, but we were unable to fully understand their origin. They can be due to optical aberrations, light source fluctuations, camera sensor temperature variations, imprecisions in the filter positions, or mispositioned ambient light shield. Yet the microscale/macroscale agreement is usually hard to reach, and the results obtained with this setup are particularly compliant in this regard.

### 2.2. Optical Model

**Figure 5:**
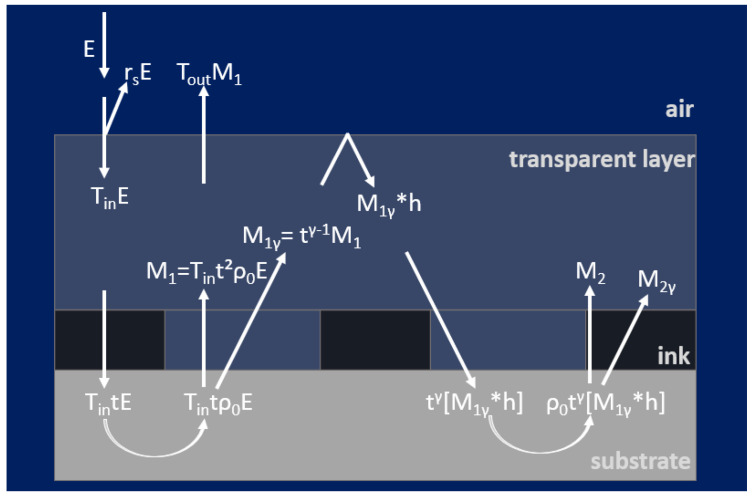
Scheme of the optical interactions within the coated halftone.

The objective is to be able to predict the reflectance of halftones when they are overlaid with a smooth transparent coating layer. An optical model has been developed and explained in [9,13,14]. It is based on the simulation of light propagation within air, coating layer, ink, and paper. It is adapted here to microscopic halftones subject to dot gain and of ink-layer thickness no longer negligible with respect to the coating layer thickness. The light interactions and equations at the origin of the model are displayed in Figure 5.

The coating is a smooth clear layer in optical contact with the printed support, which is supposed to be perfectly diffusing, i.e., Lambertian. Let us illuminate the coating layer of optical index *n_1_* with a uniform irradiance *E* at normal incidence. Light is partially reflected by the air-coating interface to a proportion denoted *r_s_*. The light transmitted through the interface, a proportion *T_in_*, transits through the transparent layer and reaches the inked support. It can then be partially absorbed by inked areas or transmitted through it to a proportion denoted *t*, the intrinsic transmittance, which is spatially and spectrally dependent of the halftone. At position (*x*,*y*) on the halftone and at wavelength λ, it is equal to:

(5) $$t\left(x,y,\lambda \right)=\sqrt{\frac{\rho \left(x,y,\lambda \right)}{\rho_{0}\left(\lambda \right)}}$$

where *ρ* is the spatial and spectral intrinsic reflectance of the halftone, and *ρ_0_* is the spectral intrinsic reflectance of the substrate, averaged over a small area. Intrinsic reflectance represents the light reflected by a material regardless of its interface with air. For a halftone, which has the same optical index as the coating layer, *n*_1_ *=* 1.5, the intrinsic reflectance derives from the Williams-Clapper reflectance model [8] and is equal to:

(6) $$\rho \left(x,y,\lambda \right)=\frac{R_{NC}\left(x,y,\lambda \right)-r_{s}}{T_{in}T_{out}+r_{i}\left[R_{NC}\left(x,y,\lambda \right)-r_{s}\right]}$$

*R_NC_* denotes the reflectance of the non-coated halftone, *T_out_* is the light proportion which is transmitted by the material-air interface –it is the same whether the material is the halftone or the coating layer–, and *r_i_* is the light proportion which is internally reflected by the material-air interface. *r_s_*, *r_i_*, *T_in_*, and *T_out_* depend on the material’s optical index and on the measurement geometry. They can be derived from the Fresnel reflectance and transmittance at the interface [29]. *ρ*_0_ derives from the same formula as *ρ.*

As the printed support and the coating layer have generally similar optical indices and are in optical contact, the interface between them has no optical effect and light travels from one to the other along straight lines. After being partially absorbed by the ink layer, light reaches the paper and is reflected into a proportion $\rho_{0}$, the same in all directions since the substrate is a Lambertian reflector. 

The light emerging from the substrate along the normal of the print is absorbed into a proportion *t* and emerges from the halftone with an exitance *M*_1_ (dependency on wavelength will be omitted in the following equations):

(7) $$M_{1}\left(x,y\right)=T_{in}t^{2}\left(x,y\right)\rho_{0}E$$

The light emerging from the substrate with a high angle of incidence travels a longer path in the absorbing ink layer than light with a small angle of incidence, which results in a higher absorption by the ink layer. This is taken into account by introducing a free parameter *γ* representing the ratio between the high incidence path length and the normal incidence path length. The intrinsic transmittance then becomes *t^γ^*, according to Beer’s Law. The exitance for light emerging with a high angle of incidence is then:

(8) $$M_{1}{}_{\gamma }\left(x,y\right)=T_{in}t^{1+\gamma }\left(x,y\right)\rho_{0}E=t^{\gamma -1}M_{1}\left(x,y\right)$$

**Figure 6:**
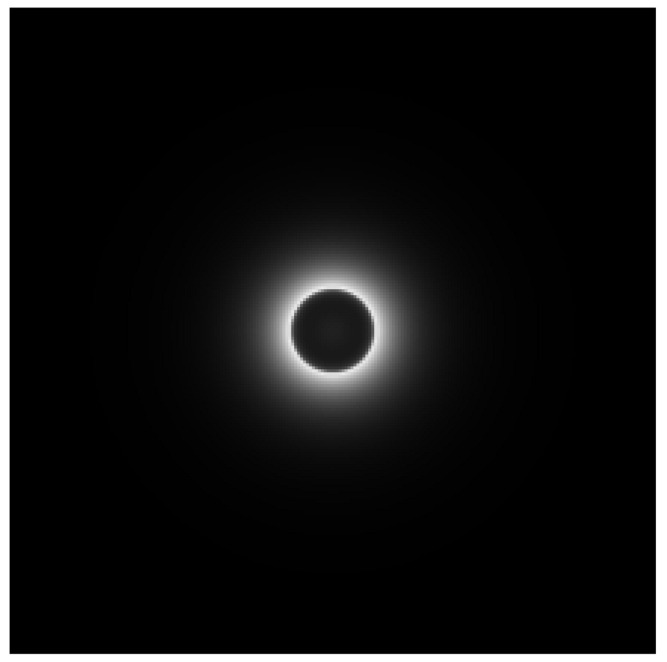
Halo function.

The light emerging from the substrate and reaching the coating-air interface is either transmitted through the interface to an extent *T_out_* and can be captured by the sensor, or it is reflected towards the halftone to an extent *r_i_.* Yet, this reflection on the coating-air interface induces a specific halo shape which has an impact on the spatial and overall reflectance of the coated halftone. This halo shape is due to the Fresnel reflectance at the interface: if light rays reach the interface with a small angle of incidence, Fresnel reflectance is low, and light is transmitted through the interface. If light rays reach the interface with an angle higher than the critical angle equal to arcsin(1/*n*_1_), the Fresnel reflectance is 1 and light is completely reflected back towards the substrate. The critical angle is 42° for the coating-air interface. The irradiance on the halftone issued from the halo effect is mapped in Figure 6.

The dark disk in the middle of the halo pattern corresponds to a low irradiance of the substrate produced by the small amount of light internally reflected by the interface at law incidence angles. The bright ring is produced by the rays totally reflected by the interface because their incidence angle is higher than 42°. The ring has a diameter $\Phi =\frac{4d}{\sqrt{n_{1}^{2}-1}}$, with *d* the thickness of the coating layer, and fades in a cos^4^ similarly to the irradiance of a small extended light source on a plane. This halo is described by a function *h*:

(9) $$h\left(x,y\right)=\frac{4d^{2}R_{n_{1},n_{0}}\left(\arctan\left[\frac{\sqrt{x^{2}+y^{2}}}{\left(2d\right)}\right]\right)}{\pi {\left(x^{2}+y^{2}+4d^{2}\right)}^{2}}$$

where $R_{n_{1},n_{0}}$ is the Fresnel reflectance at the coating-air interface, and *d* is the thickness of the coating layer. 

It is verified that light is reflected at the interface to the same extent with the halo function as with *r_i_*:

(10) $$\int_{-\infty }^{\infty }\int_{-\infty }^{\infty }h\left(x,y\right)dxdy=r_{i}$$

Most of the light coming back towards the substrate has an angle of incidence higher than 42°, it is thus subject to high absorption by the ink dots. 

The light reflected towards the halftone after reflection on the coating-air interface and absorption by the ink layer produces an irradiance *E_1_* of the substrate in each point (*x*,*y*)

(11) $$E_{1}\left(x,y\right)=t^{\gamma }\left(x,y\right)\left[M_{1}{}_{\gamma }*h\right]\left(x,y\right)$$

where symbol ∗ denotes the 2D spatial convolution operator.

After reflection on the substrate, the exitance along the normal of the print is:

(12) $$M_{2}\left(x,y\right)=\rho_{0}t^{\gamma +1}\left(x,y\right)\left[M_{1}{}_{\gamma }*h\right]\left(x,y\right)$$

*M*_2*γ*_, the exitance at a high angle is

$$M_{2}{}_{\gamma }\left(x,y\right)=t^{\gamma -1}\left(x,y\right)M_{2}\left(x,y\right).$$

It produces again a halo reilluminating the substrate, and so on. The successive exitances $M_{k}\left(x,y\right)$ satisfy the recursive equation:

(13) $$M_{k}\left(x,y\right)=\rho_{0}t^{\gamma +1}\left(x,y\right)\left[M_{k-1}{}_{\gamma }*h\right]\left(x,y\right)=\rho_{0}t^{\gamma +1}\left(x,y\right)\left[t^{\gamma -1}M_{k-1}*h\right]\left(x,y\right)$$

We can show that beyond *k =* 10, the exitance $M_{k}\left(x,y\right)$ is close to zero and the total exitance which is the sum of all exitances $M_{k}\left(x,y\right)$, can be approximated by the corresponding truncated sum. Therefore, the radiance $L\left(x,y\right)$ observed from a certain direction, after crossing the interface (factor *T_out_*), is given by:

(14) $$L\left(x,y\right)=\frac{1}{\pi }T_{out}\overset{10}{\underset{k=1}{\sum }}M_{k}\left(x,y\right)$$

The spatial reflectance factor of the coated print is the external reflectance *r_s_* of the upper interface—equal to zero in the specular excluded configuration—, plus the internal contribution given by the spatial value of radiance $L\left(x,y\right)$, divided by the radiance *1/π* scattered by a perfect white diffuser in same direction and under unit irradiance [30]:

(15) $$R\left(x,y\right)=r_{s}+лL\left(x,y\right)=r_{s}+T_{out}\overset{10}{\underset{k=1}{\sum }}M_{k}\left(x,y\right)$$

The underlaying assumptions under the use of the intrinsic reflectances in the model, Equation (5), is that the light propagation inside the substrate, causing optical dot gain, does not vary whether the substrate is coated or not. In other words, it implies that the interface between the halftone and air has no impact on the substrate point spread function (PSF), and that halo effect and dot gain are two independent phenomena. This is a strong assumption but some more complex simulations including the paper PSF that we have tested have not shown any improvement over the hereby presented model. 

### 2.3. Experimental Method and Computation

To observe the effect of the coating layer and test the optical model, an experiment has been conducted on various halftones. The spectral reflectance of the halftone samples was measured before and after applying the coating layer, and the thickness of the coating layer was evaluated. From the non-coated halftone reflectances and the layer thickness, the model can be calibrated to predict the reflectance of the coated halftones, which can then be compared to the experimental ones. 

To evaluate both the spectral and the spatial performances of the model, various halftones were designed with Adobe Photoshop^®^: two samples were composed of 8 patches of various periods made of an alternance of white and colored lines, respectively, magenta and cyan. The nominal periods of the lines were: 0.169, 0.254, 0.339, 0.423, 0.508, 0.847, 1.270 and 2.117 mm (corresponding to, respectively, 150, 100, 75, 60, 50, 30, 20, and 12 lpi), and the nominal ink surface coverage was 0.5. Pictures of the halftones are displayed in Section 3. Two additional patches were measured, corresponding to the substrate without ink and the substrate fully covered by ink (surface coverage 0, respectively, 1). The first one is needed to calibrate the model. The samples were printed on white coated paper (90 g/m^2^, Bekk smoothness ~2000 s) with an electrophotographic printer (Xerox versant 180, toner references 006R01643 for cyan and 006R01644 for magenta). The coated paper had the L*a*b* coordinates: L* = 95, a* = 1.4, b* = −4.5, measured on a white background with a 45°:0° spectrophotometer in M1 condition with D50 illuminant, which corresponds to the PS1 (Premium coated) of ISO 12647-2: 2013. The printer resolution was 1200 dpi, and the print resolution was 600 dpi. The halftone generation on Adobe Photoshop^®^ was conducted using the bitmap mode and a line threshold matrix. Any automatic color management was disabled. The halftones were printed through the Xerox Freeflow Software in direct CMJN printing mode. The electrophotographic printing process was selected to ensure that the colorant, i.e., the toner, did not penetrate the substrate, which could lead to complex light-matter interactions.

The spatial microscopic reflectances of the halftones were captured with the custom-built multispectral microscope previously detailed. It has a geometry 0°:0°, specular component excluded. Halftones were captured in HDR to ensure that both inked and non-inked areas were non-saturated. The exposure time for each filter ranged from 0.4 s to 30 s depending on the optical filter and on the color of the halftone.

For the coating process, the samples were laminated with a bright transparent foil theoretically 25 µm thick, with the laminator DRY 350 W from RBS. The lamination foil was composed of OPP and was considered perfectly transparent (its intrinsic transmittance was above 0.993). The temperature of the laminator was 110° and a coating speed of approximately 0.9 m/min was chosen. The silicon lamination rollers were 0.04 mm apart. To determine the thickness of the coated foil, thickness measurements were conducted on the prints before and after coating. The measurements were made with a micrometer from Adamel Lhomargy (m120, 15 mm diameter probe). Each patch was measured 3 times, the average thickness over each patch was used to initialize the optical model. 

After the coating step, reflectances of the samples were measured again with the microscope. Each patch, coated and non-coated, was also measured twice with the spectrophotometer CM-2600d from Konica Minolta, 8 mm aperture, with a di:8° geometry and with a UV filter to prevent fluorescence.

Afterwards, the same experiment was enlarged to more complex halftones: a third sample was designed, composed of cyan, magenta, yellow and black inked dots arranged in a rosette pattern. Each ink had a nominal surface coverage of 0.25. Two patches were made with this pattern with different periods. 

The magenta and multicolored halftone reflectances were measured by setting the sample over a white support, and the cyan halftone reflectances were measured over a black support. Even if the support had an impact on the reflectance measurement due to the transparency of the paper, it did not seem to impact the quality of the predictions.

The microscope induces spherical and chromatic aberrations which slightly blur the edges of the images. In the computation, microscope images of the line halftones were then cropped to an integer number of periods at the center of the image. One image was the exception, the coated magenta 1.270 mm-period line halftone (patch 7), which was cropped to the edge of the image due to experimental difficulties further detailed in the discussion section. The images of the multicolored halftone prints were also cropped to the center of the images but still contained enough printed dots so that cropping has no impact on the average reflectance. The image sizes were in the range of 1728 × 302 pixels and 1728 × 1533 pixels, with 13 wavelengths.

Pixels discarded during calculations because they were saturated or had an outranged reflectance represented an average of 0.12% of the pixels in the images, with high variations according to the samples and to the optical filters. The value reached an average of 1.3% for the worst sample, and 7.0 × 10^−5^% for the best sample.

The model was calibrated by measuring spatial reflectances, *R_NC_*, of the non-coated halftones and of the bare substrate, with which intrinsic reflectances were determined through Equation (6). Halo function *h* of the model was implemented through a 501 × 501 matrix of pixel pitch equal to the pixel pitch of the microscope images. It was calculated for each patch with the same coating thickness as the measured one. The size of the halo matrix was limited to enable the calculations of the 2D convolutions. The equality (10) is important for the accuracy of the model: the limited size of the halo matrix induced a small error which was compensated by adding a small scalar to all points of the halo matrix. The implemented *h* is presented in Figure 6. Parameter *γ* was fitted with experimental values of the magenta halftones with a model accounting for 1 spatial dimension giving similar results as the 2D model on line halftones. *γ* was found to be 1.23. The same value was used for the other halftones. The computations lasted approximately 70 seconds per patch.

This computation enables to compare the reflectances given by the model in Equation (15) with the spatial reflectances of the coated halftones measured with the multispectral microscope. 

## 3. Results

In order to compare predicted and measured reflectances of laminated halftones, the average spectral reflectance over an integer number of periods was evaluated, as well as the average spatial profile of the line halftones.

**Figure 7:**
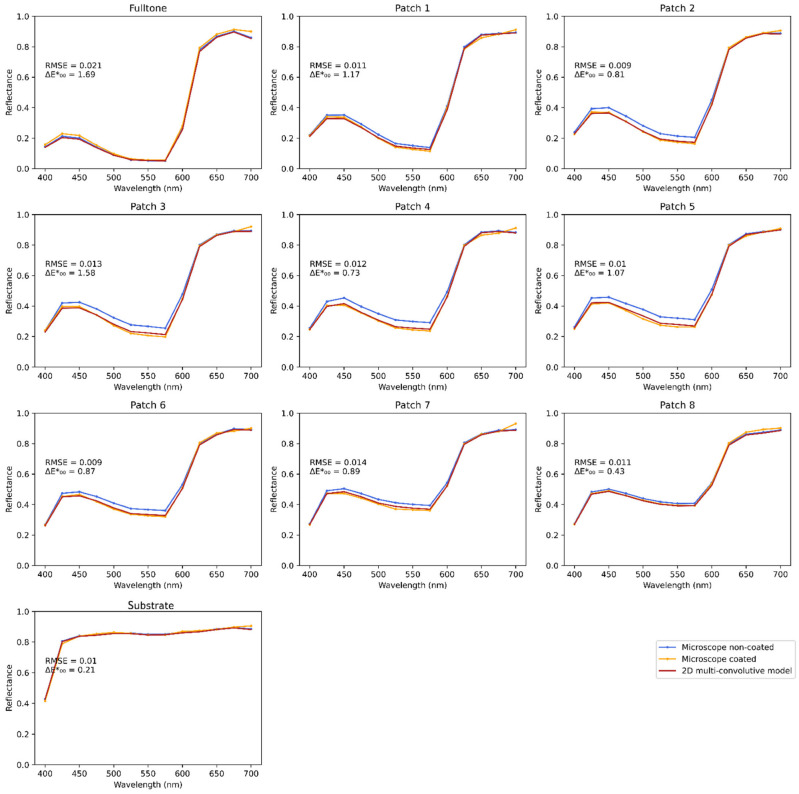
Spectra of the magenta line halftones, averaged over the spatial dimension. Patch 1 to 8 have a period of respectively: 0.168, 0.252, 0.337, 0.421, 0.505, 0.839, 1.263, and 2.104 mm. A specular component of 0.04 is added to all spectra.

Figure 7 presents the average spectral reflectances of the magenta line halftones over the area of the pictures. Experimental coated and non-coated spectra show differences due to the coating layer, especially for halftones of medium period. For these halftones, the coating layer enables light to transit from non-inked areas to magenta areas and be absorbed by them, which darkens the macroscopic reflectance of the coated copies. The fulltone and bare substrate have the same reflectance whether coated or not as their color is uniform at the microscopic scale; the convolution of a constant spatial function with the halo pattern still gives a constant spatial function, which means that the halo has no impact. The smallest-period halftone, patch 1, also shows small differences between coated and non-coated halftone reflectances. A hypothesis, which can be confirmed through the spatial characterization, is that it undergoes high optical and physical dot gains, leading to a high effective surface coverage, less distinct white lines, and a more uniformly colored halftone than patch 2 for instance. As the color is more uniform, the coating layer has less effect on the halftone. The thickest-period halftone, patch 8, is composed of lines so large that light can almost not transit from one colored area to another one, thus the coating layer has few impacts on its reflectance. 

The optical model gives a prediction of the reflectance of the coated halftones. On each graph are displayed the root mean square error between the predicted spectrum and the experimental one, as well as the color difference with the $\Delta E_{00}^{*}$ determined with the 2° observer, illuminant D65, and a perfect reflector as white reference. There is a good match between the predictions given by the model and the experimental coated-halftone reflectances, which validates the model in the spectral dimension.

**Figure 8:**
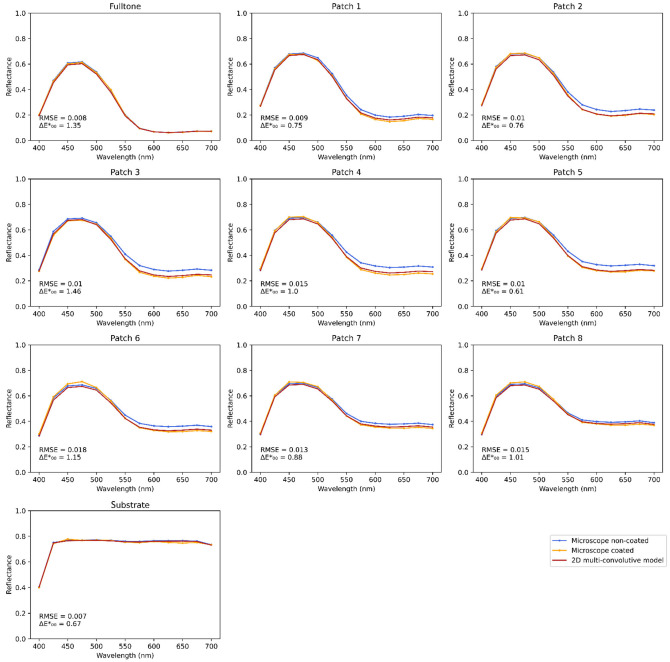
Line cyan halftone reflectance spectra, averaged over the spatial dimension. Patch 1 to 8 have a period of 0.168, 0.250, 0.334, 0.420, 0.504, 0.841, 1.259, and 2.104 mm, respectively. A specular component of 0.04 is added to all spectra.

Experiment on cyan line halftones displays similar results, presented in Figure 8.

**Figure 9:**
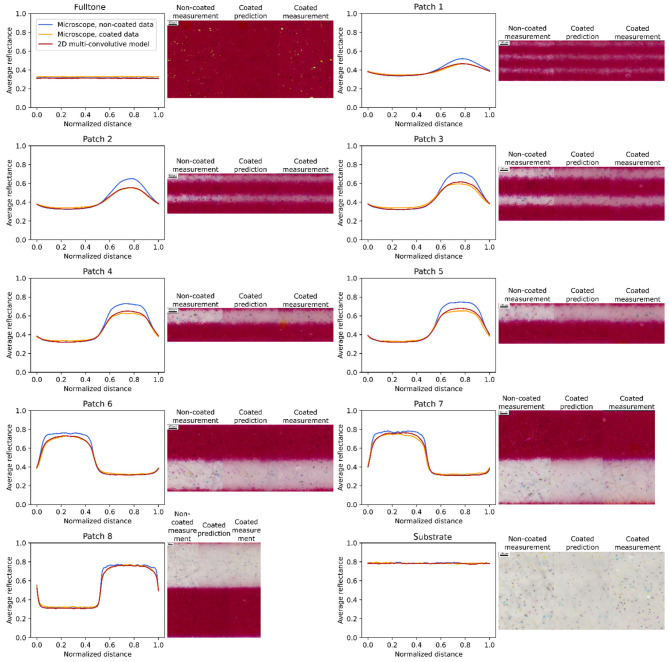
Magenta halftone reflectance profiles averaged over the spectral dimension. On their right, corresponding sRGB pictures of the non-coated, predicted, and coated halftones. Patch 1 to 8 have a period of 0.168, 0.252, 0.337, 0.421, 0.505, 0.839, 1.263, and 2.104 mm, respectively.

**Figure 10:**
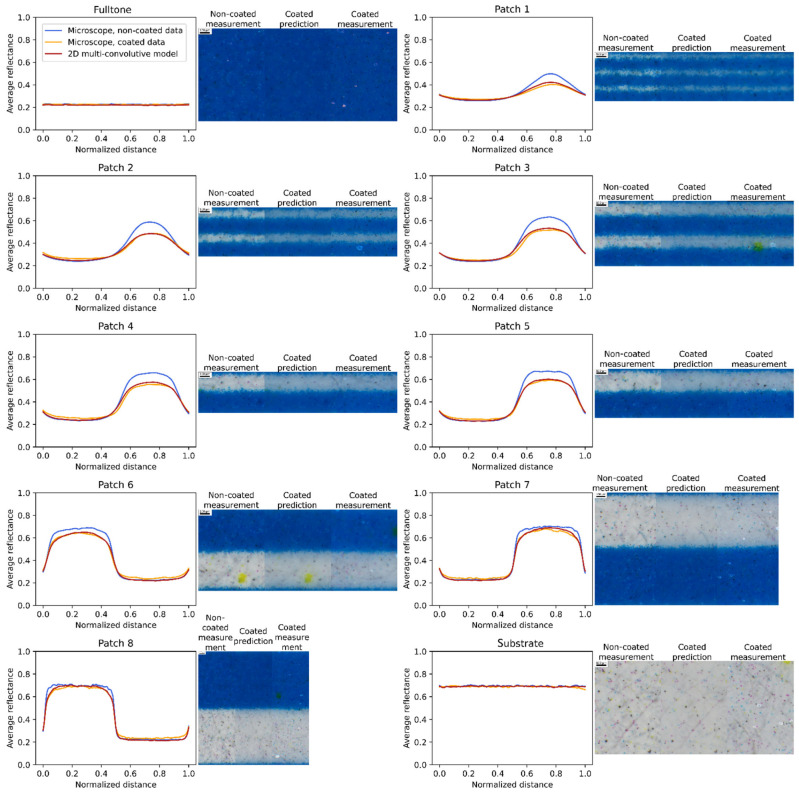
Cyan halftone reflectance profiles averaged over the spectral dimension. On their right, corresponding sRGB pictures of the non-coated, predicted, and coated halftones. Patch 1 to 8 have a period of 0.168, 0.250, 0.334, 0.420, 0.504, 0.841, 1.259, and 2.104 mm, respectively.

Thanks to the multispectral microscope setup, spatial reflectances can also be studied. They are presented in Figure 9 for magenta halftones and Figure 10 for cyan halftones. In these figures, the averaged profiles of the average spectral reflectance over the cropped multispectral images are displayed. The corresponding sRGB pictures are displayed at their side, the non-coated halftone images on the left, the predictions given by the model in the middle and coated halftone images on the right. sRGB were calculated with the norm described in [31] and images were cropped to zoom in the center of the images. These pictures allow to visually assess the measurements and predictions of spatial and spectral reflectances. Small bright yellow dots in the magenta fulltone reveal that some pixels of this image were saturated during the measurement.

In the figures displaying the reflectance profiles, Figure 9 and Figure 10, the low reflectance values correspond to the inked areas, and the high ones to the non-inked areas. The profiles are computed from the microscopic image, by taking all the profile attached to all the vertical lines and averaging them. Since the printed lines are composed of small ink dots also called ‘satellites’ (see the microscopic pictures in Figure 9 and Figure 10), the average profile is not perfectly crenel-shaped but a little smoothened. Moreover, the well-known phenomenon of optical dot gain due to light scattering by the support behind the ink patterns also contributes to blur the boundaries of the inked area, and thereby to smoothen the profile discontinuities. It is particularly emphasized with high frequency halftones (thin lines, e.g., patches 1 and 2) where non-inked lines appear slightly magenta as light can easily travel from the inked lines to the non-inked ones. This results in halftone patterns looking more homogeneous, less contrasted, and reflectance profiles with a lower amplitude. With low frequency halftone (patches 7 and 8), the optical dot gain is visible through the subtle color gradient next to the border of the inked area. All these effects are much more pronounced when the halftones are coated, since the coating layer induces an additional optical dot gain. For high frequency halftones (patches 1 and 2), the microscopic pictures look even more homogeneous, and the amplitude of reflectance profiles even reduced. For low frequency halftone (patches 7 and 8), the color gradient next to the border of the ink areas is magnified; the discontinuities in the reflectance profiles are more smoothened than in absence of coating. The change of amplitude of the reflectance profiles due to the coating varies according to the contrast of the non-coated halftone patterns and, therefore, to the halftone frequency; with high frequency halftones, whose contrast before coating is lower (consequence of the optical dot gain induced by the diffusing support), the coating effect is less pronounced. 

Figure 9 and Figure 10 also show the reflectance profiles predicted by the optical model (from images of the non-coated patches), which can be compared with the ones measured with the microscope (images of the coated patches). The agreement between the predicted and measured profiles is fairly good. This is particularly remarkable as the non-coated and coated measurements were not done in the same area of the halftones, which proves the robustness of the method. There remains for some patches a small difference in the amplitudes of the profiles: an error of 5% is observed in the brightest areas of patch 4 and 5, whereas the error is less than 2% for patches 2 or 6. This might be due to the heterogeneities of the printed lines, or to differences between the properties of the real materials and the ones assumed by the model, for instance scattering inks or a not perfectly smooth coating layer. 

The performance of the model can also be visually assessed in the sRGB pictures. The color difference between the non-coated and coated patches is striking, whereas the predicted and measured images of the coated patches look very similar. A closer look simply shows that the predicted images are more blurred, as a consequence of the multiple convolution process operated on the images of the non-coated patches, than the measured images of the coated patches. 

**Figure 11:**
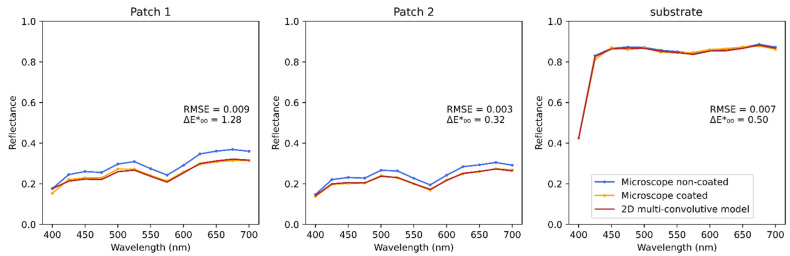
Average spectra of the multi-ink dot halftones, and of the bare substrate.

**Figure 12:**
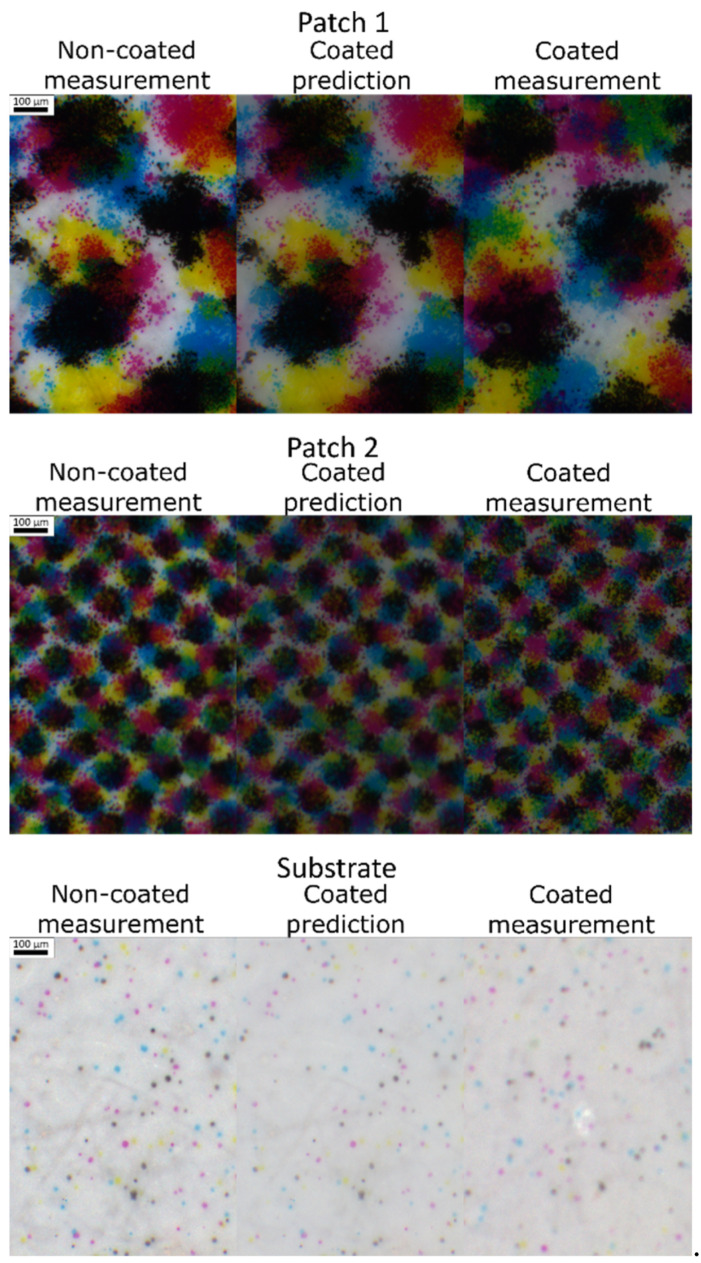
sRGB pictures of the multi-ink dot halftones: non coated on the left, predicted in the middle, and coated on the right.

The case of the single-ink line halftones that we have developed in detail above was useful to analyze the predictive performance and the imperfections of the model, both spectrally and spatially, in the simplest cases. However, the method applies with any kind of halftone pattern. We propose to illustrate its capacities through the example of two gray color patches, printed with four-ink clustered dot halftones. The average spectra are presented in Figure 11, and the sRGB pictures in Figure 12. In these figures can be seen the good agreement between experiment and predictions, especially with patch 2 for which the ΔE*_00_ is only 0.3 unit, and the color appearance in the predicted and measured images for the coated patch look very similar.

The performances of the microscope measurement system and of the model are summarized in Table 1. The performances of the model are also displayed when the parameter γ is not fitted, i.e., equal to 1. The results are further discussed in Section 4.

## 4. Discussion

The rather good agreements between the predictions and measurements obtained with our method throughout our experiments tend to confirm that we have well identified the optical phenomenon in play when a clear layer is coated on top of a halftone print, and well described it in our optical model. We can also consider that the assumptions on which the model relies, regarding both the structure of the halftone print and the optical properties of the materials, reasonably meet the reality in rather conventional printing configurations. Some disadvantages and sources of errors, however, can be mentioned. 

Spectral microscopic imaging is not usual in the printing domain, which often prefer spectral-only measurements with a spectrophotometer. Furthermore, spectral microscopes are not common instruments on the marketplace, especially with the spectral and spatial resolution that we have here. In our case, the microscope has been extended to spectra measurements by our own means. The spectral resolution is particularly critical for the optical model to give a good accuracy: a minimum step size of 25 nm, consistent over the whole spectrum, is required. The calibration of the microscope is a delicate operation that requires special caution. Regarding the spectral aspect, the difficulty comes from the fact that the exposition time of the camera must be adapted to each filter in order to guarantee a good SNR and prevent saturation of the sensor in some pixels, despite the HDR algorithm used in the acquisition process. The homogeneity of the white standard used as reference should be checked, and its spectral reflectance should be precisely known. We took care to verify that the average reflectance measured by the microscope and those measured by a spectrophotometer satisfying the recommendations by the CIE for colorimetric studies are consistent with each other, to ensure that the calibration was correctly performed. The difference in illumination and observation geometry between the two systems can cause large deviations with some types of surfaces, but the risk was limited with the surfaces studied here since the diffusing background was almost Lambertian and the specular component was excluded. We have achieved a color difference between the spectra from the two systems of ΔE*_00_ = 1.0 on average, which is already a point of satisfaction. On the spatial aspect, the main issue was the focus, since the overlay is transparent. The focusing operation was done separately on each measured sample. Since the acquisition was rather long, around 10 min for one sample due to the multiple acquisitions required by the HDR process, slight vibrations around the microscope could lead to a loss of focus, or a blurring effect. Note that microscopes may have some chromatic aberrations at the borders of the images, and the spectral values can be significantly impacted. It is, therefore, preferable to analyze the center of the images. 

Regarding the printed samples, the Xerox printer that we used generates some yellow dots which cannot be avoided as they are a signature of the printer. One is visible on the patch 6 of the non-coated cyan halftones. Most of the time, we could take the microscopic pictures in areas without these yellow dots. In some cases, however, due to the presence of theses yellow dots at the center of the image, we had to analyze the border of the image impacted by the chromatic aberration (a slight violet tint can be seen at the frontier between inked and non-inked areas, for example in patch 7 of the coated magenta halftones). 

In the model, the value of the parameter γ, related to average path length of the light within the coating layer, can be discussed. In theory, it should be around 1.23, and all our predictions have been made with this value. However, we wanted to test the prediction accuracy when its value is 1 (or, equivalently, this parameter γ is removed from the equations). The result, detailed in Supplementary Materials, is that the results are as good as with γ = 1.23. The average $\Delta E_{00}^{*}$ between the predictions and the measurements was 0.92 ± 0.39 with γ = 1.23, and 0.99 ± 0.40 with γ = 1. This parameter may become important if the thickness of the absorbing ink layer is high in proportion to the thickness of the coating layer. Its fitting may also account for complex interactions between the optical dot gain of the substrate and the interreflection inside the coating layer, two phenomena which are considered independent in the model.

Finally, regarding the comparison between the predicted and measured data, we could use the standard color distance metrics proposed by the CIE for comparing spectral reflectances, namely the ΔE*_00_ metric. We did not try to compare quantitatively the color images, but simply display them to let the observer appreciate their similarity visually.

## 5. Conclusions

The change of color of a halftone print when applying a clear coating on top of it is easy to observe and well known in printing workshops. However, the physical reason for this change has been discovered only recently: it is due to the multiple reflection process between the diffusing support and the coating-air interface, where each point illuminated on the support generates a ring-shaped halo that meets neighboring inked area and increases the chance for light to be absorbed. This optical effect, that can be qualified as a dot gain effect, of course depends on the spatial distribution of the inks according to the screening used to generate the halftone colors. For the first time, we could predict the spectral reflectance of multi-ink halftone prints after coating, by taking a microscopic spectral image of the prints before coating and applying an optical model describing the multi-convolutive optical process. The color prediction accuracy is rather good, and we could also render rather well the blurring effect induced by the coating on the contours of the ink areas. This paper describes the optical model and the experimental setup used. The calibration of the spectral microscope, the most delicate step in our protocol, was checked by comparing the average spectral reflectance computed over a spectral image and the spectral reflectance measured with a standard spectrophotometer for color applications satisfying the recommendations by the CIE. The rather good agreement between the two measurement systems enabled to validate the microscopic measurements (of resolution 1.386 µm per pixel), and to overcome the challenge of linking microscale measurements with macroscale measurements.

The method could find some promising applications for a better color management of coated or laminated prints. It could also be used for the thickness measurement of thin transparent layers, by depositing it on a known halftone pattern and analyzing the color variation, or generate innovative anti-counterfeiting features in the security printing domain.

## Figures and Tables

**Table 1:** Performances of the measurement system and of the optical model.

**Magenta Samples**					
**Patch #**	**Coating Thickness (µm)**	**Halftone Period (mm)**	ΔE*_00_		
			**Spectrophotometer vs. Microscope, Non-Coated Samples**	**Model vs. Experiment, Coated Samples, with γ = 1.23**	**Model vs. Experiment, Coated Samples, with γ = 1**
Fulltone	20.3		0.44	1.69	1.20
1	21.0	0.168	2.01	1.17	1.40
2	19.7	0.252	1.24	0.81	1.14
3	19.7	0.337	1.19	1.58	1.81
4	19.3	0.421	0.68	0.73	1.16
5	19.7	0.505	0.75	1.07	1.43
6	19.7	0.839	0.44	0.87	0.95
7	20.3	1.263	0.89	0.89	1.00
8	21.0	2.104	0.28	0.43	0.33
Substrate	20.0		1.51	0.21	0.21
Average	20.1		0.94	0.94	1.06
Standard deviation	0.6		0.55	0.46	0.48
**Cyan Samples**					
**Patch #**	**Coating Thickness (µm)**	**Halftone Period (mm)**	ΔE*_00_		
			**Spectrophotometer vs. Microscope, Non-Coated Samples**	**Model vs. Experiment, Coated Samples, with γ = 1.23**	**Model vs. Experiment, Coated Samples, with γ = 1**
Fulltone	23.3		1.27	1.35	0.45
1	23.0	0.168	1.82	0.75	1.12
2	23.3	0.250	0.86	0.76	0.48
3	24.7	0.334	0.92	1.46	1.31
4	23.0	0.420	0.85	1.00	1.40
5	23.3	0.504	0.93	0.61	1.01
6	24.7	0.841	1.24	1.15	1.24
7	23.0	1.259	0.94	0.88	0.95
8	24.0	2.104	0.73	1.01	0.80
Substrate	23.7		1.16	0.67	0.69
Average	23.6		1.07	0.96	0.94
Standard deviation	0.6		0.32	0.29	0.33
**Multicolored Samples**					
**Patch #**	**Coating Thickness (µm)**		ΔE*_00_		
			**Spectrophotometer vs. Microscope, Non-Coated Samples**	**Model vs. Experiment, Coated Samples, with γ = 1.23**	**Model vs. Experiment, Coated Samples, with γ = 1**
1	22.3		0.38	1.28	1.21
2	23.7		1.85	0.32	1.08
Substrate	25.3		0.60	0.50	0.50
Average	23.8		0.94	0.70	0.93
Standard deviation	1.5		0.80	0.51	0.37
**Overall**					
	**Coating Thickness (µm)**		ΔE*_00_		
			**Spectrophotometer vs. Microscope, Non-Coated Samples**	**Model vs. Experiment, Coated Samples, with γ = 1.23**	**Model vs. Experiment, Coated Samples, with γ = 1**
Average	22.1		1.00	0.92	0.99
Standard deviation	1.9		0.47	0.39	0.40

## Data Availability

Data is contained within the article or Supplementary Material.

## Supplementary Materials

The following supporting information can be downloaded at https://www.mdpi.com/article/10.3390/jimaging8090243/s1, Figure S1: Spectra of the magenta line halftones, averaged over the spatial dimension. Patch 1 to 8 have a period of 0.168, 0.252, 0.337, 0.421, 0.505, 0.839, 1.263, and 2.104 mm, respectively. A specular component of 0.04 is added to all spectra. The model predictions are evaluated with γ = 1; Figure S2: Line cyan halftone reflectance spectra, averaged over the spatial dimension. Patch 1 to 8 have a period of 0.168, 0.250, 0.334, 0.420, 0.504, 0.841, 1.259, and 2.104 mm, respectively. A specular component of 0.04 is added to all spectra. The model predictions are evaluated with γ *=* 1; Figure S3: Magenta halftone reflectance profiles averaged over the spectral dimension. Patch 1 to 8 have a period of 0.168, 0.252, 0.337, 0.421, 0.505, 0.839, 1.263, and 2.104 mm, respectively. The model predictions are evaluated with γ *=* 1; Figure S4: Cyan halftone reflectance profiles averaged over the spectral dimension. Patch 1 to 8 have a period of 0.168, 0.250, 0.334, 0.420, 0.504, 0.841, 1.259, and 2.104 mm, respectively. The model predictions are evaluated with γ *=* 1; Figure S5 Average spectra of the multi-ink dot halftones, and of the bare substrate. The model predictions are evaluated with γ *=* 1.

## References

[1] Kipphan H. (2001). Handbook of Print Media.

[2] Kokla V. (2021). Assessing Historical Printed Materials Using the Combination of Historical Information and Imaging Techniques. Case Study: Greek Postcards of the Early 20th Century. Int. Circ. Graph. Educ. Res..

[3] Childers A., Etheredge A., Flannery S., Freeman J. (2008). Effects of Varnish on Printed Material.

[4] Cigula T., Hudika T., Donevski D. (2021). Color Reproduction on Varnished Cardboard Packaging by Using Lower Ink Coverages Due to the Gray Component Replacement Image Processing. Color. Res. Appl..

[5] Galić E., Ljevak I., Zjakić I. (2015). The Influence of UV Varnish on Colorimetric Properties of Spot Colors. Procedia Eng..

[6] Hudika T., Majnarić I., Cigula T. (2020). Influence of the Varnishing “Surface” Coverage on Optical Print Characteristics. Tehnički Glasnik.

[7] Sakovic B. Effect of Different Types of Lamination on Colour Gamut and Tone Value Increase of Digital Prints. Proceedings of the 2nd International Student Conference “Printing Future Days 2007”.

[8] Williams F.C., Clapper F.R. (1953). Multiple Internal Reflections in Photographic Color Prints. J. Opt. Soc. Am..

[9] Simonot L., Hébert M., Gerardin M., Monpeurt C., Fournel T. (2018). Halo and Subsurface Scattering in the Transparent Coating on Top of a Diffusing Material. J. Opt. Soc. Am. A.

[10] Simonot L., Hébert M. (2018). Un Étonnant Halo Lumineux. Microscoop.

[11] Cornu A. (1890). Sur Le Halo Des Lames Épaisses, Ou Halo Photographique, et Les Moyens de Le Faire Disparaitre. J. Phys. Theor. Appl..

[12] Dolin L.S. (2019). Laser Bathymetry Based on the Halo Effect. Appl. Opt. AO.

[13] Hébert M., Dailliez F., Simonot L. (2021). Why a Clear Coating Modifies Halftone Color Prints. Electron. Imaging.

[14] Dailliez F., Hébert M., Blayo A., Chagas L., Fournel T. (2021). Impact of a Transparent Coating on the Reflectance of a Line Halftone Pattern. Coatings.

[15] Callahan F.P. (1952). Light Scattering in Halftone Prints. J. Opt. Soc. Am. JOSA.

[16] Geoffrey R., Olympe C., Thierry F., Mathieu H. (2019). Measurement of the Diffusion of Light within Paper. J. Opt. Soc. Am. A.

[17] Gustavson S. (1997). Dot Gain in Colour Halftones.

[18] Rogers G. (1997). Optical Dot Gain in a Halftone Print. J. Imaging Sci. Technol..

[19] Yang L., Lenz R., Kruse B. (2001). Light Scattering and Ink Penetration Effects on Tone Reproduction. J. Opt. Soc. Am. A JOSAA.

[20] Clapper F.R., Yule J.A.C. (1953). The Effect of Multiple Internal Reflections on the Densities of Half-Tone Prints on Paper. J. Opt. Soc. Am. JOSA.

[21] Yule J.A.C., Nielsen W.J. (1951). The Penetration of Light into Paper and Its Effect on Halftone Reproduction. Proc. TAGA.

[22] (2021). Picture Copyright Images & Rêves. Alpes Is Here.

[23] Vallat-Evrard L. (2019). Mesure, Analyse et Modélisation à l’échelle Microscopique de Points Imprimés Pour Améliorer Les Solutions de Lutte Anti-Contrefaçon.

[24] Finlayson G., Zhu Y. An Improved Optimization Method for Finding a Color Filter to Make a Camera More Colorimetric. Proceedings of the Electronic Imaging.

[25] Imai F. Multispectral Image Acquisition and Spectral Reconstruction Using a Trichromatic Digital Camera System Associated with Absorption Filters. Proceedings of the International Symposium on Multispectral Imaging and Color Reproduction for Digital Archives.

[26] Parmar M., Imai F., Ho S., Farrell J. A Database of High Dynamic Range Visible and Near-Infrared Multispectral Images. Proceedings of the SPIE—International Society for Optical Engineering.

[27] Imai F., Berns R. Spectral Estimation Using Trichromatic Digital Cameras. Proceedings of the International Symposium on Multispectral Imaging and Color Reproduction for Digital Archives.

[28] Brydegaard M., Merdasa A., Jayaweera H., Ålebring J., Svanberg S. (2011). Versatile Multispectral Microscope Based on Light Emitting Diodes. Rev. Sci. Instrum..

[29] Kriss M. (2015). Handbook of Digital Imaging.

[30] Nicodemus F.E., Richmond J.C., Hsia J.J., Ginsberg I.W., Limperis T. (1977). Geometrical Considerations and Nomenclature for Reflectance.

[31] SRGB. https://www.color.org/chardata/rgb/srgb.xalter.

