# Supplementary material for: Use of Multispectral Microscopy in the Prediction of Coated Halftone Reflectance
Source: J Imaging. 2022 Sep 8;8(9):243. doi: 10.3390/jimaging8090243 (PMC9504082; doi:10.3390/jimaging8090243)
Supplement: Supplementary file 1 [file jimaging-08-00243-s001.zip › jimaging-1841565-supplementary.pdf]

The figures displayed in this section aim at presenting the performances of the 2D multi-convolutive model when the value of the parameter γ, related to average path length of the light within the coating layer, is 1 (or, equivalently, when this parameter γ is removed from the equations). Figures S1, S2 and S5 present the predictions of the spectral reflectances, with $\gamma = 1$, of respectively the magenta, cyan, and multi-ink halftones. Figures S3 and S4 present the profile predictions, with $\gamma = 1$, of respectively the magenta and cyan halftones.

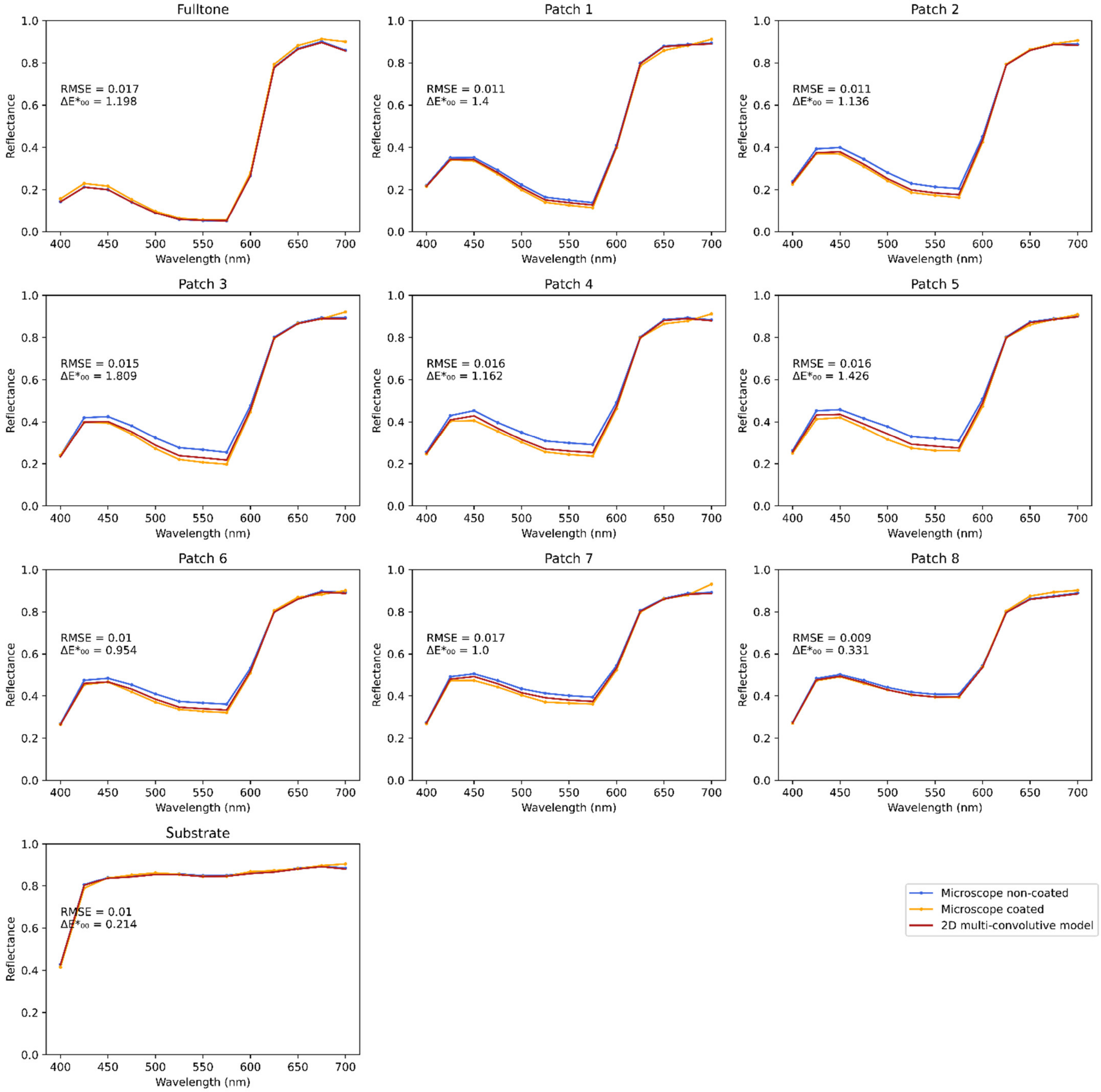

Figure S1. Spectra of the magenta line halftones, averaged over the spatial dimension. Patch 1 to 8 have a period of 0.168, 0.252, 0.337, 0.421, 0.505, 0.839, 1.263, and 2.104 mm, respectively. A specular component of 0.04 is added to all spectra. The model predictions are evaluated with $\gamma = 1$.

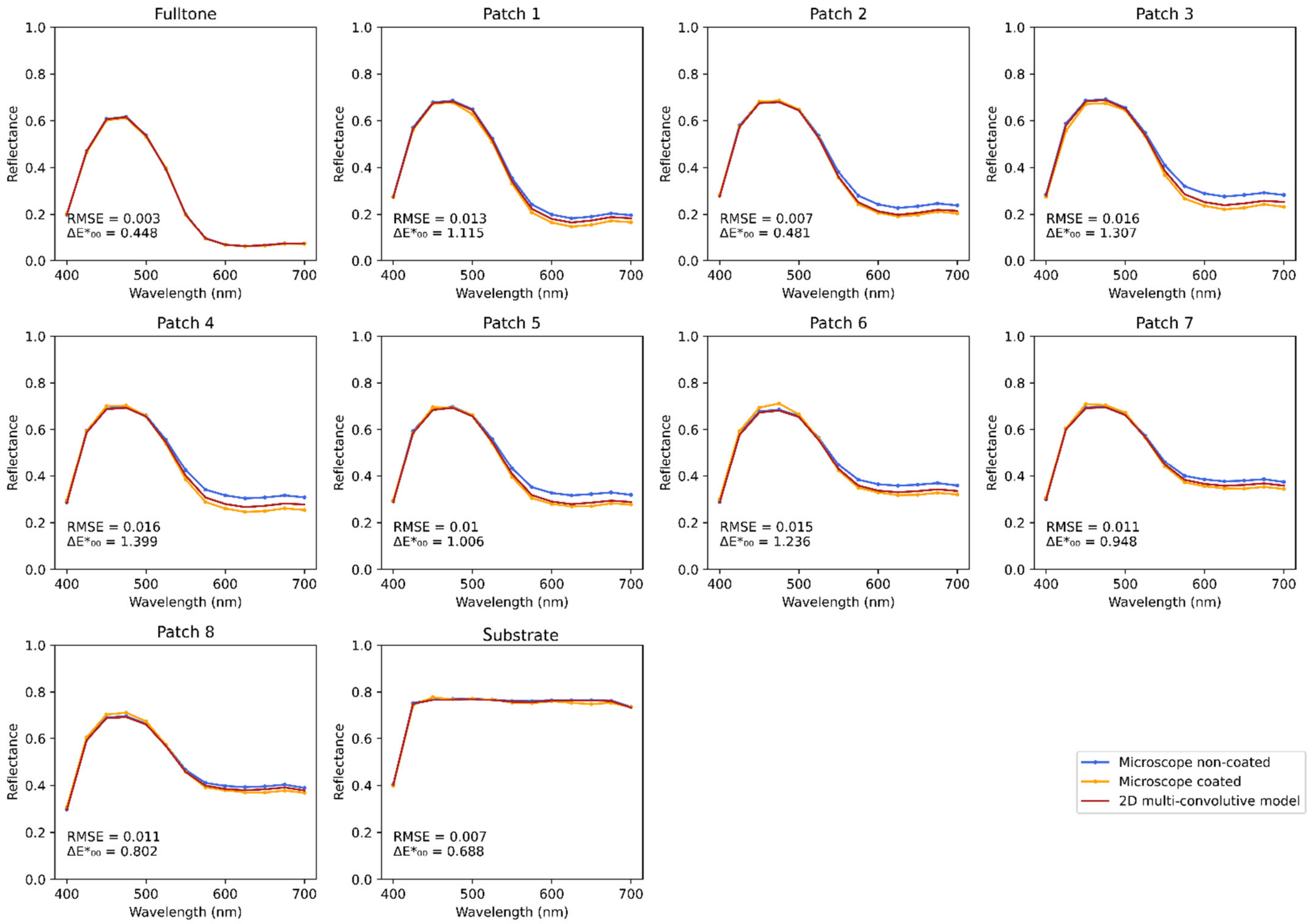

Figure S2. Line cyan halftone reflectance spectra, averaged over the spatial dimension. Patch 1 to 8 have a period of 0.168, 0.250, 0.334, 0.420, 0.504, 0.841, 1.259, and 2.104 mm, respectively. A specular component of 0.04 is added to all spectra. The model predictions are evaluated with $\gamma = 1$.

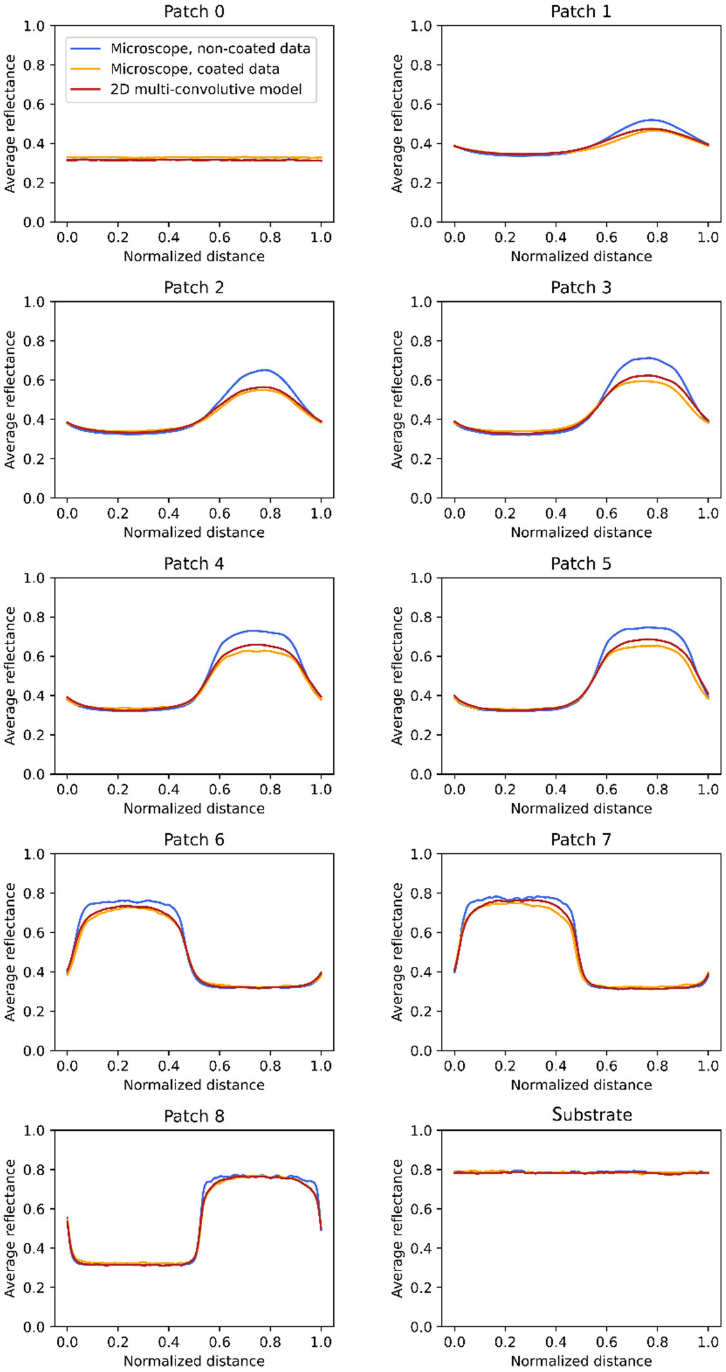

Figure S3. Magenta halftone reflectance profiles averaged over the spectral dimension. Patch 1 to 8 have a period of 0.168, 0.252, 0.337, 0.421, 0.505, 0.839, 1.263, and 2.104 mm, respectively. The model predictions are evaluated with $\gamma = 1$.

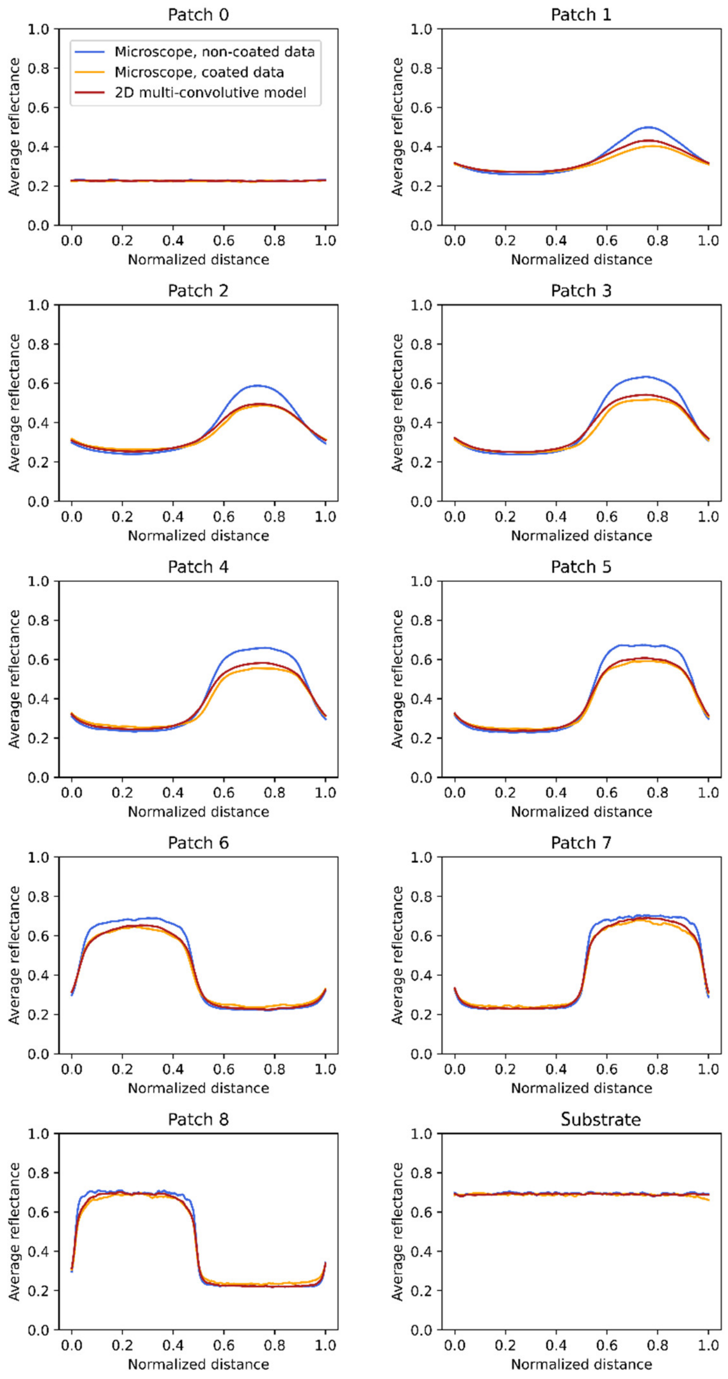

Figure S4. Cyan halftone reflectance profiles averaged over the spectral dimension. Patch 1 to 8 have a period of 0.168, 0.250, 0.334, 0.420, 0.504, 0.841, 1.259, and 2.104 mm, respectively. The model predictions are evaluated with $\gamma = 1$.

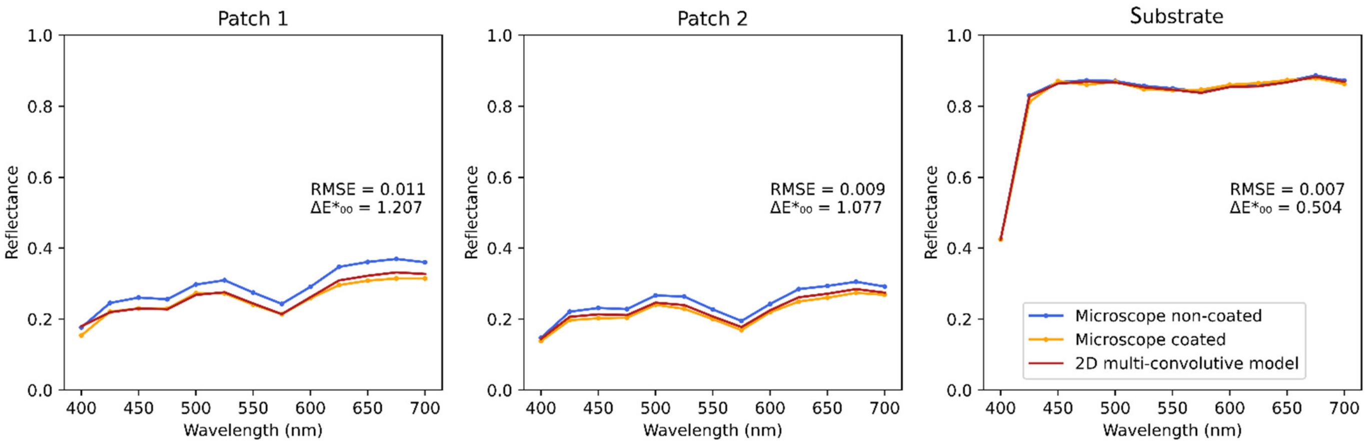

Figure S5. Average spectra of the multi-ink dot halftones, and of the bare substrate. The model predictions are evaluated with $\gamma = 1$.
